# Design and Fabrication of a High-Speed Atomic Force Microscope Scan-Head

**DOI:** 10.3390/s21020362

**Published:** 2021-01-07

**Authors:** Luke Oduor Otieno, Bernard Ouma Alunda, Jaehyun Kim, Yong Joong Lee

**Affiliations:** 1 Department of Mechanical Engineering, Kyungpook National University, Daegu 41566, Korea; luke.otieno@gmail.com; 2 School of Mines and Engineering, Taita Taveta University, P.O. Box 635, Voi 80300, Kenya; benalunda10@gmail.com; 3 Department of Mechanical and Design Engineering, Hongik University, Sejong 30016, Korea; kimj@hongik.ac.kr

**Keywords:** atomic force microscopy, high-speed atomic force microscope, high-speed atomic force microscope scan-head

## Abstract

A high-speed atomic force microscope (HS-AFM) requires a specialized set of hardware and software and therefore improving video-rate HS-AFMs for general applications is an ongoing process. To improve the imaging rate of an AFM, all components have to be carefully redesigned since the slowest component determines the overall bandwidth of the instrument. In this work, we present a design of a compact HS-AFM scan-head featuring minimal loading on the Z-scanner. Using a custom-programmed controller and a high-speed lateral scanner, we demonstrate its working by obtaining topographic images of Blu-ray disk data tracks in contact- and tapping-modes. Images acquired using a contact-mode cantilever with a natural frequency of 60 kHz in constant deflection mode show good tracking of topography at 400 Hz. In constant height mode, tracking of topography is demonstrated at rates up to 1.9 kHz for the scan size of 1μm×1μm with 100×100 pixels.

## 1. Introduction

In atomic force microscopy (AFM), scanning speed is affected by all components of the instrument. Although the improvement in the scanning speed of the AFM started soon after the instrument was invented [1], the progress in the development of high-speed AFM (HS-AFM) had been slow due to the innovations needed in the electronic, optical, and mechanical products and processes required for designing faster instruments [2]. One of the earliest realizations of HS-AFM occurred nearly two decades ago [3,4]. Since then, further advances have been made to improve the scan-head and other HS-AFM components and methods [5,6,7,8,9,10,11,12,13,14,15,16,17].

When one aspect of HS-AFM is improved, usually one or more of other aspects of the instrument requires further improvements. For example, the improved mechanical bandwidth of scanners and cantilevers usually calls for faster drive amplifiers, feedback components, cantilever deflection detection electronics, cantilever excitation, and so forth [6,13]. Small, high-frequency cantilevers have become commercially available, and along with this improvement comes the requirement for redesigning the AFM scan-head with optical elements capable of focusing a laser spot on the small cantilever area. Small cantilevers are desirable in high-speed scanning applications since for the same spring constant, reducing the cantilever size increases their resonance frequency, leading to faster maximum image acquisition rates. As an alternative to the small cantilevers required for high-speed imaging, another approach enhances the stiffness of large active cantilevers using force feedback in order to achieve high-speed imaging of large biological samples [18]. Q-control of active cantilevers has also been used to improve imaging rates in tapping-mode AFM [19,20]. Cantilever Q-control does not necessarily call for head redesign to achieve high-speed imaging. In general, however, the development of HS-AFM heads is usually directed towards using small cantilevers [4,6,7,13,21].

Some of the main considerations that constrain the mechanical design of a HS-AFM scan-head are the optical architecture to be used and the scanning technique to be adopted. The selection of the optical architecture depends on the cantilever deflection detection technique to be employed. Owing to its cost and simplicity of implementation, optical beam deflection (OBD) is the most widely adopted technique. With OBD, two optical architectures can be used to separate the incident beam of light from the reflected beam of light: spatial separation architecture and polarization-based separation architecture [6,22,23]. The choice of one architecture or the other also depends on, among other things, the scanning technique to be employed. Of utmost importance in any OBD system is the ability of effective optical beam tracking with minimal errors, even for samples with large vertical and lateral features.

HS-AFM designs typically employ one of three major scanning techniques: sample scan, tip scan, and combined tip-sample scan. In sample scan HS-AFMs, the sample is mounted on an integrated XYZ-scanner [4,6,13,23]. The advantage of this method is that it simplifies the overall mechanical design of the head and tracking of the optical beam. However, this approach limits the specimen size because of the limited dimensions of the sample stage usually mounted on top of a small high-speed Z-scanner. Sample scan HS-AFMs cannot be trivially integrated with other optical microscopy techniques [24]. Additionally, designing an integrated XYZ-scanner is more challenging than designing separate scanners. Despite the mentioned disadvantages, sample scan HS-AFM designs are the most widely reported in literature because of their practicality.

Tip scan HS-AFMs have a stationary sample, and a cantilever is attached to an integrated XYZ scanner [25,26]. This approach makes it easier to integrate the HS-AFM with an optical microscope, but it makes tracking of the optical beam cumbersome. It also limits the achievable lateral scan rates as well as the scan range. Holding the cantilever securely on top of the Z-scanner while still maintaining a mechanical bandwidth high enough for high-speed imaging also becomes a non-trivial task and significant effort has been put into providing solutions to this problem [24]. Tip-sample scan HS-AFMs combine tip scanning with sample scanning [12,27]. This approach separates the design of the XYZ-scanner into two (a lateral and a vertical scanner) or three (a one dimensional scanner for each axis) separate scanners, thereby simplifying the design process of the scanners. While this configuration allows the possibility for the examination of large specimens by using large-range sample stages as compared to sample scan designs, optical beam tracking still presents a challenge.

A common tip-sample scan approach typically comprises of the XY-sample scan and Z-tip scan, where the XY- and Z-scanners are separated [12,28]. The XY-scanner has the sample stage while the Z-scanner has the cantilever mounted onto it. However, a more recent approach uses separate X-, Y- and Z-scanners and then connects the Z-scanner mounted with a cantilever holder to the Y-scanner in-order to facilitate the tip scan [27]. Separating the design of the three scanner axes improves their individual dynamic performance. However, since the Y- and the Z-scanners are mechanically coupled, cross-coupling between the Y and the Z axes is unavoidable. Additionally, the extra load on the Y-scanner constrains its design. For both tip-sample scan designs, an appropriate optical architecture is required to simplify optical beam tracking. In the design reported in Reference [12], the employed optical architecture is not immediately apparent. In the work reported by Liu and his co-workers [27], a simple laser tracking method that takes into account the movement of the tip along the Y and Z axes during scanning is reported. However, the plane of the cantilever is tilted relative to the focal plane of the focusing lens. This inevitably leads to OBD errors associated with the displacement of the Z-scanner [28]. In general, while combined tip-sample scan architectures offer attractive advantages to HS-AFM designers, optical beam tracking and appropriate cantilever holding mechanisms are problems that need to be addressed.

To the best of our knowledge, combined tip-sample scan designs presented in literature also do not incorporate photothermal cantilever drive. Miyata et al. [12] only performs imaging in contact-mode while Liu et al. [27] excites the cantilever using a piezoelectric actuator (PEA). Problems with piezoelectric actuation include reduced stability when used for long periods at a time and distortion of amplitude and phase curves when used in liquid environments [29,30,31]. Photothermal cantilever excitation overcomes these problems and therefore it is becoming common to implement it in HS-AFM scan-heads [13].

This work presents our contribution towards the effort to develop a combined tip-sample scan HS-AFM with a simple optical beam tracking and photothermal drive. We adopt the combined XY-sample scan and Z-tip scan approach in order to constrain the optical beam tracking to the Z-direction. The optical axis is tilted from the vertical to place the cantilever on a plane normal to the incident optical beam [32]. The arrangement reduces the OBD errors that would emanate from large displacements by the Z-scanner. To separate the XY- and the Z-scanners, a cantilever-mounted Z-scanner is attached onto the AFM scan-head’s optics block. We design a collar (which we refer to as the Z-collar) for mounting both the Z-scanner and a compact aspheric lens for focusing the laser beam onto the cantilever. The Z-collar can be easily dismounted from the scan-head to facilitate a cantilever exchange and/or focusing. We also incorporate the photo-thermal drive capability to potentially provide stable dynamic mode imaging in air and liquid [30]. We then use homemade XY-scanners and a custom-programmed controller to evaluate the high-speed imaging capability of the developed system. Images of Blu-ray disk tracks are acquired in contact- and tapping-modes.

Compared to sample scan HS-AFMs presented in References [3,13], our work separates the XY-scanner from the Z-scanner, making the scanner design process simpler. In addition, the XY-scanners can easily be changed to accommodate different imaging requirements. Tip scan HS-AFMs also use combined XYZ-scanners in addition to fairly cumbersome laser tracking systems as the cantilever moves laterally and vertically [25,26]. Other than simplifying the design of the scanners by separating the XY- and the Z-scanners, our work also simplifies laser tracking by constraining the movement of the cantilever along the vertical dimension only. Reducing laser tracking complexity also increases the potential scan range of our system compared with tip scan HS-AFMs. Combined tip-sample scan approaches reported in References [12,27] do not integrate photothermal cantilever drive that can potentially be used for stable tapping mode imaging in all environments. Also, unlike in References [12,27], the cantilever plane is normal to the incident beam to potentially reduce OBD errors emanating from the displacement of the Z-scanner during imaging [28].

## 2. Scan-Head Design

We set out to design a HS-AFM scan-head that incorporates a cantilever mounted Z-scanner in order to separate the lateral and vertical scan axes. Additional considerations were general compactness of the design, relative ease of cantilever replacement, and similar ease of use compared to commercial AFMs. We modeled the design using a 3D CAD software (Inventor, Autodesk, San Rafael, CA, USA), then machined and assembled mechanical parts with optical parts. During the experiments, the entire HS-AFM scan-head is mounted on a 1-inch optical post (SRP-35, Lee Optics, Sejong, Korea) by using a mounting bracket that fits into a dovetail subassembly. The HS-AFM scan-head can easily be dismounted from the optical post for cantilever exchanges, focus adjustment, and general servicing.

### 2.1. Optical Design

**Figure 1:**
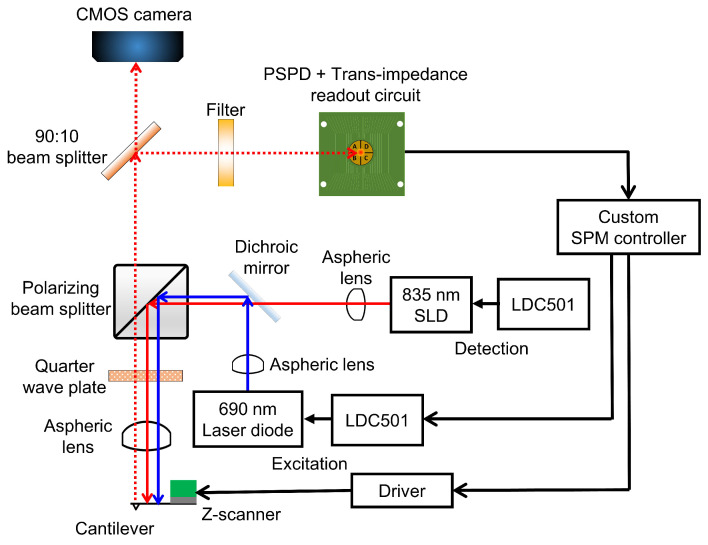
Schematic of the optical architecture of the proposed design. All components are contained in the scan-head except the laser diode controllers, the scanning probe microscope (SPM) controller, deflection signal readout circuit and the piezo driver.

The HS-AFM scan-head design adopted polarization optics to achieve the cantilever excitation and deflection detection [22,33]. Figure 1 shows the optical architecture of our design. The cantilever deflection is detected by using an 835 nm superluminescent diode (SLD) (SLD-380-MP-TO9-PD, Superlum, Carrigtwohill, Ireland). A SLD is chosen because its light has a shorter coherence length than lasers, thus reducing the risk of light interference when performing measurements on reflective samples [34]. The excitation of the cantilever is accomplished by using a 30 mW 690 nm laser diode (HL6738MG, Thorlabs, Newton, NJ, USA). The wavelengths of the detection SLD light and the excitation laser should be close to minimize the focal shift between the two wavelengths [33]. The aspheric lens (A397-B, Thorlabs) used in this work leads to a focal shift of about 85 μm between the two wavelengths. The focusing challenge that is presented by this focal shift is overcome by first focusing the detection light, then adjusting the focus of the excitation laser and the detection light within the focal depth of the detection light. Using a 785 nm excitation laser instead, for instance, reduces the focal shift to about 25 μm. However, the optical filter (Hoya IR-80, Edmund Optics, Barrington, NJ, USA) used in this work to block the excitation laser from reaching the detection photodiode allows a significant portion of 785 nm laser power through to the photodiode. The transmitted excitation laser power significantly interferes with the detection of cantilever deflection. We therefore chose to use a 690 nm excitation laser diode to minimize the interference. To reduce the excitation laser power reaching the detection photodiode, an optical filter that absorbs nearly all of the excitation laser should be used.

Each diode is controlled by using a dedicated laser diode controller (LDC) (LDC501, Stanford Research Systems) capable of modulating the light power up to 1.0 MHz in the constant current mode. The light from each diode is collimated with an aspheric lens (A230-B, Thorlabs) with a high numerical aperture (NA) of 0.55 and an effective focal length of 4.51 mm. High NA ensures that more light from the source is collected. Each diode-collimator set is fixed onto a kinematic mount to provide a means for positioning the focal spot on the cantilever and the photodetector. After collimation, a long-pass dichroic mirror (DMLP805T, Thorlabs) is used to combine the paths of the two beams. The two beams are then passed through a polarizing beamsplitter (PBS) (PBS102, Thorlabs) which allows the s-polarized light to be reflected by $90^{\circ }$ towards a quarter-wave plate (QWP) (WPMQ05M-830, Thorlabs). After passing through the quarter-wave plate twice—to and from the cantilever—the s-polarized light from the PBS is rotated into p-polarized light that is transmitted through the PBS towards the detection photodiode. The incident beams pass through an aspheric lens (A397-B, Thorlabs) and are then focused onto the cantilever. The aspheric lens has a NA of 0.3 and the focal length of 11 mm to provide enough room for mounting the Z-scanner. The Z-scanner mounted with cantilever holding mechanism fits into the space between the cantilever and the focusing lens.

From the back of the cantilever, the reflected light is collimated by the aspheric lens and rotated into a p-polarized beam that gets transmitted through the polarizing beamsplitter. A second beamsplitter (BS) (BSX05, Thorlabs) reflects 90% of the reflected beam to a long-pass filter (Hoya IR-80, Edmund Optics) eventually arriving at a quadrant photodiode (QPD) (QP5-6 TO, First Sensor, Berlin, Germany). The long-pass filter has a cut-on wavelength of 800 nm. Its purpose is to filter out the excitation laser beam and to transmit most of the longer wavelength detection light. The remaining 10% of the reflected light is transmitted to a CMOS camera for viewing the cantilever and the sample during mounting, alignment, approach, scanning, and other operations. All optical components are mounted inside the scan-head.

### 2.2. Mechanical Design

**Figure 2:**
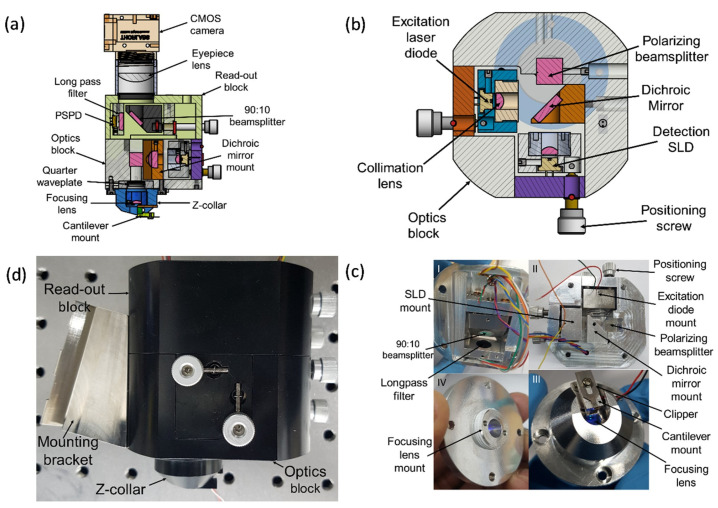
HS-AFM scan-head design. (**a**) A vertical section of 3D CAD model; (**b**) A horizontal section of the optics block; (**c**) Assembled scan-head components: I. read-out block, II. optics block, III. Z-collar, and the focusing lens mount partially mounted in the Z-collar. The focusing lens mount can be translated vertically within the Z-collar to place a focal spot on the cantilever; (**d**) Assembled scan-head with the mounting bracket fixed.

The mechanical design of the scan-head facilitates mounting of the optical elements to enable detection and/or excitation beams to be positioned onto the cantilever, the QPD, and the camera. A 3D CAD model of the mechanical design is shown in Figure 2a. The scan-head has three major subcomponents as shown in Figure 2c. The first part is the collar for mounting the focusing lens and the Z-scanner. The second part is the optics block for fixing the SLD, the laser diode, and other optical components. A horizontal section of this part is shown in Figure 2b. The third part is the read-out block for the QPD and the camera. The 90:10 beamsplitter mount is attached to a kinematic mount that facilitates positioning of the light portion reflected by the beamsplitter onto the photodiode during alignment. All parts are assembled securely together using stainless steel screws as shown in Figure 2d.

Collimation and focusing aspheric lenses are mounted in the threaded studs to provide a simple means of translation for varying the distance between the lenses and the object/source during focusing/collimation. The collimation lens mounts are screwed onto the detection and excitation diode kinematic mounts using M3 screws. The collimation mounts are also fixed onto the fabricated kinematic mounts for positioning the light beams. On the other hand, the focusing lens mount is screwed into the collar as shown in Figure 2c. Focusing is achieved by translating the lens mount vertically within the collar to adjust the distance between the lens and the back of the cantilever. The vertical translation of the focusing lens mount is achieved by rotating the lens mount clockwise or counter-clockwise within the collar. Each full rotation of the lens holder is equivalent to a vertical distance of a single thread pitch. A lens tube (SM1L10, Thorlabs) and a thread adapter (SM1A9, Thorlabs) connects the AFM scan-head to a complementary metal-oxide-semiconductor (CMOS) camera.

## 3. Scanners

**Figure 3:**
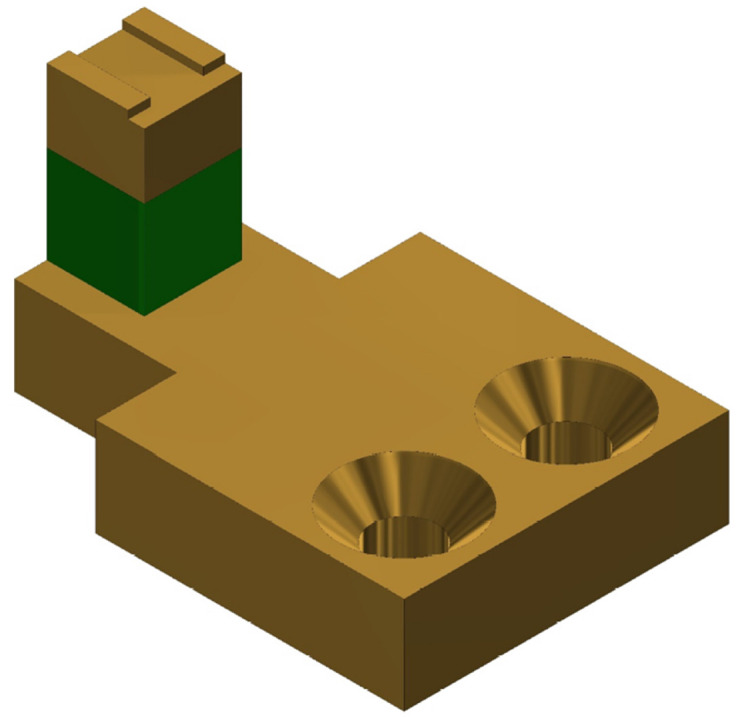
A 3D CAD model of the Z-scanner.

The Z-scanner consists of a Z-piezo mount, the Z-piezo (PA4JKW, Thorlabs), and a cantilever mount. Each part is connected to the other in the order previously listed. 3D CAD model of the scanner is shown in Figure 3. The cantilever is attached to its holder by using a glue. Using glue simplifies the design of the cantilever mount since no extra mechanical components are required to hold the cantilever securely. For similar Z-scanner designs, reduced loading on the Z-scanner increases its first resonant frequency, and this was the key reason why the use of glue was adopted. The disadvantages of using glue include increasing the likelihood of destroying cantilevers during removal, inability to reposition installed cantilevers once the glue cures, and the need to remove glue residue from the mount after every use. The cantilever mount is fabricated from aluminum to minimize the mass-loading on the Z-piezo. The Z-scanner has a travel range of approximately 2.3μm at 100 V. A separate parallel kinematic XY-scanner is also designed and fabricated. Each axis of the XY-scanner is actuated using a dedicated piezo stack (PK4FA2P2, Thorlabs). The scan range of the lateral scanner in either axis is approximately 3.3μm at 100 V. The sample stage of the developed XY-scanner is 1.2cm×1.2cm, which is still larger than typical sample stages available in HS-AFMs with combined XYZ-scan architectures reported in References [3,13]. The scan ranges of the scanners is comparable to those reported by other HS-AFM designs [7,12,13].

**Figure 4:**
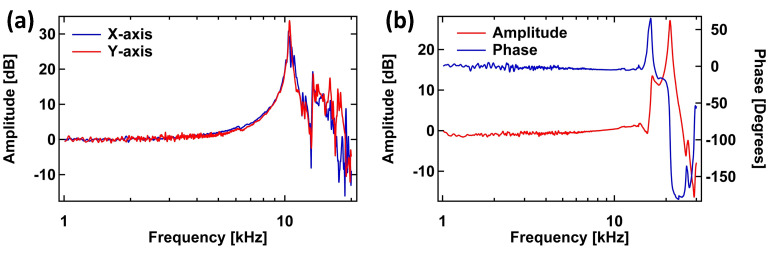
Frequency response of the (**a**) XY- and (**b**) Z-scanners.

The dynamic behavior Z-scanner was measured with a 330 kHz cantilever (RTESPA-300, Bruker, Fremont, CA, USA) in contact with a sample. The variations in the cantilever deflection caused by the driven response of the Z-scanner was then measured using a sweepable digital lock-in amplifier in a commercial SPM controller (AFT-HSC50, Anfatec, Oelsnitz, Germany). The frequency responses of the two XY-scanner axes were measured using the sweepable lock-in amplifier and a capacitive probe (ADE-5504, Microsense, LLC, Lowell, MA, USA) with its gauging module (ADE 5810, Microsense, LLC). The responses of the XY- and Z-scanners are shown in Figure 4a,b. For the XY-scanner, Figure 4a shows the first main resonance at about 10.4 kHz for both axes.

The first resonant mode of the measured Z-scanner response occurs at 16.7 kHz. While the measured frequency of the scanner’s first resonant mode is lower than previsouly reported HS-AFM Z-scanner designs [7,12,14], it is still sufficient to demonstrate high-speed scanning. Furthermore, the limitation in the bandwidth of the Z-scanner can be improved by fixing the entire base of the Z-piezo mount to the HS-AFM head. In our current design, one end of Z-piezo mount is attached to the HS-AFM head by using two screws while the piezo with the cantilever holder is glued onto the mount’s free end. The entire Z-scanner assembly is similar to a lever fixed at one end and free at the other end. Therefore, the response of the Z-scanner is dominated by the structure of the assembly, rather than the response of the loaded Z-piezo. In our next iteration of the design, we will improve on the design of the Z-scanner and its assembly with the other components of the scan-head in order to improve its mechanical bandwidth.

## 4. Imaging Experiments

**Figure 5:**
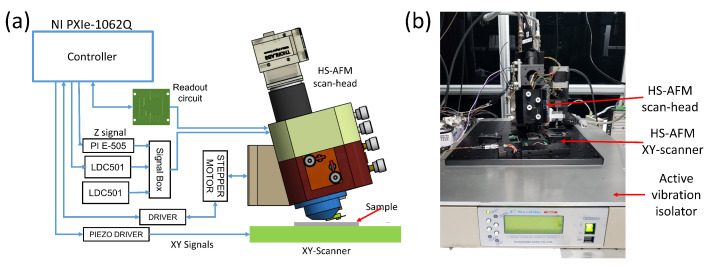
The experimental setup. (**a**) Schematic of the setup; (**b**) Photographs of the setup.

A schematic and photograph of the setup are shown in Figure 5a,b. The main components of the experiment for high-speed imaging were the custom-programmed controller, the readout circuit, the lateral scanner, the high-voltage piezo drivers, and the developed HS-AFM scan-head. We used a custom-programmed FPGA-based high-speed controller based on National Instruments hardware and software. The vertical feedback controller has a maximum sampling rate of 100 MHz while the scan engine can acquire 100×100 pixel images at the rate of 40 frames per second. The vertical feedback control is achieved by using a proportional-integral-derivative (PID) controller, and the amplitude estimation for dynamic imaging mode is achieved by using an FPGA-based coherent detector. The homemade readout circuit has the 3 dB bandwidth of approximately 8 MHz.

To drive the XY-scanner, we used a homemade dual-channel piezo driver with a maximum peak-to-peak output voltage of 290 V and the open-circuit bandwidth of 12 kHz. The driver is built with commercially available high voltage operational amplifiers (PA-85A, Apex Microtechnology, Tucson, AZ, USA). The Z-scanner is driven by a commercial amplifier (PI E-505, Physik Instrumente, Karlsruhe, Germany). The other concern to contend with is the increasing phase lag as the scan speed increases due to the limited slew rate of the amplifier. In the lateral axes, this results in mirroring of features near the left (in trace images) or the right (in retrace images) of the image [4]. In the vertical axis, the limited slew-rate of the commercial amplifier is expected to limit the vertical feedback bandwidth of the HS-AFM. The limited vertical feedback bandwidth leads to progressive blurring of topographical features in the scanned images as the scanning speed increases. Additionally, the bandwidth of our current Z-scanner is limited by the first resonant mode of the scanner at about 16.7 kHz so the benefits of using a high-bandwidth Z-scanner driver would not be realized without first improving the scanner’s control bandwidth. The limited bandwidth limits the maximum achievable imaging rate when vertical feedback controller is active, but it is still sufficient to perform high-speed imaging. With these phenomena known in advance, high-speed imaging capability of the developed HS-AFM can be carefully demonstrated.

For controlling the laser diodes, we used commercial laser diode controllers (LDC501, Stanford Research Systems) in constant-current mode to avoid the added complexity of monitoring power in constant power mode. We also automate the sample approach using a five-phase stepper motor to limit the incidences of crashing the cantilever tip onto the sample surface.

To demonstrate the use of the designed scan-head in high-speed imaging, we obtained images of Blu-ray disk data tracks at different scan rates. The XY-scanner is operated in the open-loop mode and without a compensation for hysteresis and creep. Contact-mode imaging was carried out using a triangular cantilever with a natural frequency of 60 kHz (NP-S, Bruker, Fremont, CA, USA). We monitored the ability of the AFM scan-head to track lateral features of the sample as the line scan rates increased. Images are obtained in both constant deflection and constant height modes. The constant height mode images demonstrate the possibility of acquiring images beyond the bandwidth of the vertical feedback loop [2,35]. In our current setup, the vertical feedback bandwidth is limited by the control bandwidth of the Z-scanner, which is in turn limited by the first resonance frequency of the scanner. Improving the control bandwidth of the Z-scanner should in principle improve the image acquisition rates of the system.

## 5. Discussion

**Figure 6:**
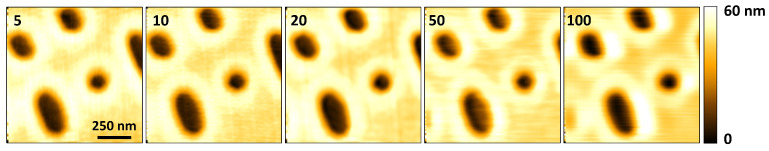
A 1μm×1μm scan of Blu-ray disk data tracks consisting of 100×100 pixels. The line rate, in Hz, is indicated in the top left corner of each image.

Figure 6 shows the results of scans obtained at 5 Hz to 100 Hz to demonstrate the ability of the scan-head to acquire images of nearly consistent quality at low speeds as well as at moderately high speeds. The images are acquired in constant deflection contact-mode.

**Figure 7:**
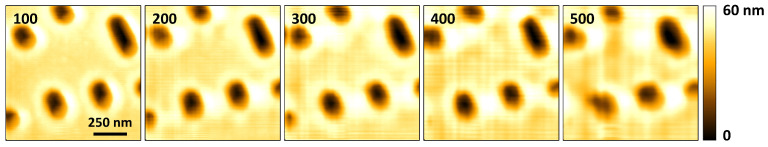
Topographical images of a 1μm×1μm Blu-ray disk scan consisting of 200×200 pixels acquired at different line rates. The line rates, in Hz, are indicated in the top left corner of each image.

To further demonstrate the capability of high-speed imaging, Figure 7 shows the topographical images of a 1μm×1μm scan of Blu-ray disc tracks acquired at 100 Hz to 500 Hz. Again, the images are acquired in constant deflection contact-mode. As the scan rate is varied, the level of deterioration in the quality of the acquired topographical images up to 400 Hz is barely noticeable. However, at 500 Hz, the change in the quality of the image is more discernible. The control bandwidth of the vertical feedback loop is no longer able to maintain cantilever deflection while tracking the topography of the sample with the desired accuracy. To improve the imaging rates in constant deflection mode, the control bandwidth of the vertical feedback loop can be improved using two main approaches: implementing control methods that account for Z-scanner oscillations [36] and/or improving the mechanical design of the Z-scanner to increase the frequency of its first resonant mode [3]. At present, we are only using a PID controller which quickly leads to instability as its parameter gains are increased to match high scan rates.

Also, as the imaging speed increases, portions of the features near the left edge of the trace image start getting mirrored. Mirroring is caused by phase lag introduced by the inertia of the lateral scanner and/or the AFM’s electronics [4,13]. The size of the mirrored portion of the image increases with increase in scan rate as shown in the images acquired at 200 Hz and 300 Hz in Figure 7. The mirroring effect can be reduced by using low phase lag electronics and by reducing the size of the load attached to the lateral scanner [4,13]. Where the feedback of the lateral scanner’s position is available, mirroring can be eliminated since mirroring is brought about by inaccurate matching of the XY-scanner position with the topographical data.

**Figure 8:**
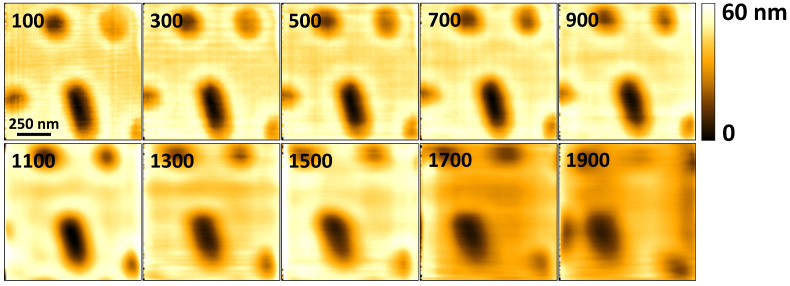
Topographical images of a 1μm×1μm Blu-ray disk scan consisting of 100×100 pixels acquired at different line rates. The line rate, in Hz, is indicated in the top left corner of each image. The images are acquired using sinusoidal raster scan waveforms to reduce the effects of XY-scanner dynamics.

Other artifacts are visible around feature edges in Figure 6 and Figure 7, and the artifacts increase in prominence as the scan speed increases. These artifacts are apparent from 100 Hz, and they appear close to the right edge of each feature. Response of the feedback controller—and the Z-scanner hysteresis—to sudden changes in topography leads to overshoot in the transition regions. As the images are acquired in trace mode, scanning the horizontal axis from left to right, areas appearing to have the highest topography next to the right edges of the features represent the overshoot. The images in Figure 8 which are acquired in constant height mode are less affected by this phenomenon because the controller is turned off. The artifacts due to sudden transitions in sample topography can be reduced by implementing improved feedback controllers instead of using a PID controller in isolation [36,37].

Another artifact that is visible in the images in Figure 6 and Figure 7 is the presence of horizontal and vertical lines in some of the images. The horizontal lines are caused by electronic noise. Filtering out the noise during post-processing of the acquired imaging data was not effective for all the the scan rates, as is visible in the images acquired from 200 Hz to 400 Hz. Computing the frequency spectrum of the acquired cantilever deflection data revealed noise components with a fundamental at 60 Hz, and multiple harmonics. Improved design of the signal acquisition system to attenuate the noise components is expected to reduce this effect. The vertical lines are due to vibrations that are caused by the (1) sharp turnaround edges of the fast-axis triangular positioning signals, and (2) positioning the scanner in steps, from one pixel to the next. As the scanning speed increases, the vertical lines become more spaced out horizontally, making them less apparent at 500 Hz in Figure 7. These effects can be reduced by appropriate shaping of positioning signals [5,11].

Additional images were acquired in constant height mode, with the vertical feedback controller disabled. The images obtained in constant height mode demonstrate the ability of the system to acquire images at rates that are unencumbered by the Z-scanner bandwidth. However, in constant height mode, the interaction between the cantilever and the sample is not controlled, and sensitive samples can easily be destroyed during imaging. The results obtained were as shown in Figure 8. The artifacts visible in these images are attributed to the dynamics of the cantilever and the lateral scanner. To further reduce the effects of XY-scanner dynamics, the images are acquired using approximations of sinusoidal scan waveforms in the fast axis. However, the scanner displacement from one point to the next is still implemented in steps, leading to harmonics that can potentially excite the scanner’s resonant modes, albeit to a lesser degree. The sinusoidal scan waveforms used to acquire the images are digitally synthesized using 198 points per waveform cycle. Each scan has an area of 1μm×1μm and a pixel size of 100×100. The constant height mode images demonstrate the ability of the system to acquire images at 1900 Hz using the contact-mode cantilever with a natural frequency 60 kHz in air. While in constant height mode the system can potentially acquire images at higher rates, beyond 1900 Hz the drive current from the amplifier is no longer sufficient to produce sinusoidal output voltages while driving the scanner. The turn-around edges of the output drive voltages become sharp again, creating harmonics that can induce larger vibrations in the lateral scanner. The results in Figure 8 show that the limitations of the vertical feedback bandwidth are the cause of considerable deterioration in image quality above a line rate of 400 Hz in the constant deflection mode images shown in Figure 7.

To demonstrate the ability of the scan-head to be used in dynamic mode imaging, scans of a Blu-ray disk were acquired in tapping-mode using a cantilever with a first natural bending frequency, $f_{0}$, of 316 kHz (RTESPA-300, Bruker, Fremont, CA, USA). The cantilever is photothermally excited by modulating the excitation laser power. The mean power for the excitation laser is 10 mW. The modulation depth is about 50% (10 mW peak to peak). Cantilever oscillation amplitude is estimated once every 10μs. The Q-factor of the cantilever’s free resonance curve was estimated at 360 in air. The high Q-factor value limits the cantilever’s imaging bandwidth, $B\approx \frac{\pi f_{0}}{Q}$, in tapping-mode, to an approximate value of 2.7 kHz [38]. In this case, the slow response of the cantilever becomes the limiting factor in the vertical feedback loop.

**Figure 9:**
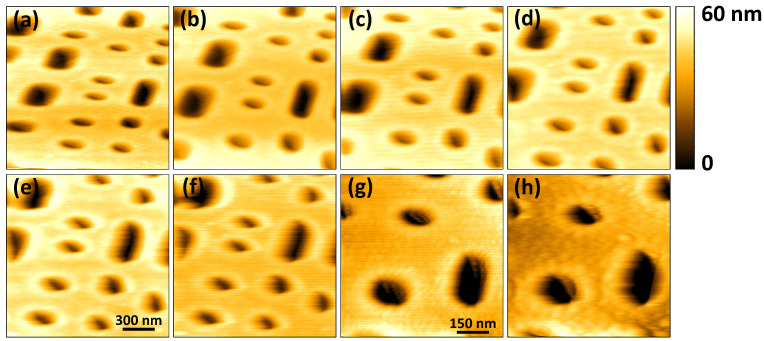
Topographical images of a Blu-ray disk sample consisting of 256×256 pixels acquired at different line rates. The scan area is 1μm×1μm for images (**a**–**f**) and 0.75μm×0.75μm for images (**g**,**h**). The scan rates in Hz are (**a**) 2; (**b**) 5; (**c**) 10; (**d**) 20; (**e**) 40; (**f**) 50; (**g**) 50, and (**h**) 100.

Figure 9 shows the images acquired in tapping-mode at different line rates. At 50 Hz, the deterioration in the quality of the acquired image is visible. When the scan area is reduced, the reduction in probe-sample velocity leads to improvement in the acquired image quality at 50 Hz, as shown in Figure 9f,g. In Figure 9h, acquired at 100 Hz, further deterioration in the image quality is noticeable. The line rates are limited by the slow response of the cantilever in tapping-mode. Faster imaging rates can be achieved in tapping-mode by using a high-frequency cantilever with a low Q-factor or by the artificial reduction of the cantilever’s Q factor by the Q-control technique [3,19].

In Figure 9, the horizontal and vertical lines are not visible in the images. The horizontal lines have disappeared because the lock-in detection method used to estimate the amplitude of vibration of the cantilever filters out the electronic noise. Additionally, each line is composed of 256 pixels as opposed to 100 pixels used for the images in Figure 6, Figure 7 and Figure 8. Since the same area is scanned, increase in the number of pixels reduces the step sizes as the XY-scanner moves from one pixel location to the other. This in turn reduces the likelihood of exciting undesired lateral scanner vibrations. The imaging rates are also fairly low, reducing the risk of vibrations induced by triangular scan waveform.

Ultimately, the extent to which a HS-AFM is applicable is dependent on its ability to acquire images from samples that can easily be damaged or distorted by probe-sample interactions in less than ideal environments. In a number of studies, HS-AFMs are developed for biological applications [3,6,13]. Biological samples are challenging to image because they are usually contained in liquid cells and they can easily be damaged if probe-sample interaction is not carefully controlled. By contrast, a Blu-ray disk sample is considerably easy to image using a HS-AFM. It is typical to use high frequency cantilevers to perform high-speed imaging of soft biological samples in tapping-mode. However, we note that if the optical design is able to focus excitation and detection lasers appropriately, selecting small high frequency cantilevers can enable imaging of soft samples in tapping mode at high speeds. Our configuration can potentially be optimized to create focused spot diameters, $w_{o}=\frac{2\lambda }{\pi \mathrm{NA}}$, that are small enough to be used with some small high-speed cantilevers [21,33]. Other than the laser spot size, other requirements for high-speed imaging may be met by adjusting feedback controller designs [39], and implementing advanced tapping-mode techniques [14], for greater control of probe-sample interactions. For samples that are less sensitive to mechanical damage, larger high-speed tapping-mode cantilevers can be used with the system as is [27]. In addition, our system implements photothermal cantilever excitation that has been demonstrated to work reliably in challenging imaging environments and over large excitation frequency ranges, as opposed to the widely used piezoelectric cantilever excitation [30]. We have not demonstrated the imaging of challenging samples with our system. However, we believe that with minor modifications, our system can potentially be used for high-speed imaging of a sample with a sensitive surface in a liquid environment.

## 6. Conclusions

In this work, we have developed a tip-sample scan HS-AFM scan-head. For the presented design, the procedures for loading cantilevers and focusing are relatively simple once all the components of the scan-head have been assembled. Also, the cantilever is placed in the focal plane of the focusing lens in order to reduce the problems associated with laser tracking. We have demonstrated the capability of high-speed imaging in contact- and tapping-modes. In tapping-mode, the high Q-factor of the cantilever has limited the line rate to about 100 Hz for a scan area of 0.75μm×0.75μm. However, we believe that much faster rates can be attained by using tapping-mode cantilevers with high imaging bandwidth. In constant deflection mode, the imaging rate of our instrument is limited to around 400 Hz by the bandwidth of the Z-scanner while in constant height mode tracking of the sample topography has been obtained at 1900 Hz for an image size 100×100 pixels and a scan area of 1μm×1μm. Improving the control bandwidth of the Z-scanner would potentially improve the constant deflection imaging rates of our current HS-AFM setup in the future.

## Data Availability

Data sharing is not applicable to this article.

## Abbreviations

The following abbreviations are used in this manuscript:

AFM	Atomic Force Microscope
BS	Beamsplitter
CAD	Computer-Aided Design
CMOS	Complementary Metal-Oxide-Semiconductor
HS-AFM	High-Speed Atomic Force Microscope
LDC	Laser Diode Controller
NA	Numerical Aperture
OBD	Optical Beam Deflection
PBS	Polarizing Beamsplitter
PEA	Piezoelectric Actuator
PID	Proportional-Integral-Derivative
QPD	Quadrant Photodiode
QWP	Quarter-Wave Plate
SLD	Super Luminescent Diode
SPM	Scanning Probe Microscope
